# 

**Published:** 1840

**Authors:** 


### THE MEDICO-CHIRURGICAL REVIEW,

AND

### JOURNAL

OF

### PRACTICAL MEDICINE.

*(NEW SERIES.)*

VOLUME THIRTY-THREE,

[1st of APRIL, to 30th of SEPTEMBER,]

1840.

VOL. XIII. of DECENNIAL SERIES.

EDITED

BY JAMES JOHNSON, M.D.

PHYSICIAN EXTRAORDINARY TO THE LATE KING,

AND

HENRY JAMES JOHNSON, ESQ.

LECTURER ON ANATOMY AT THE SCHOOL OF ST. GEORGE'S HOSPITAL IN KINNERTON STREET.

Surgeon Gen'l's Office LIBRARY 57473

LONDON:

PUBLISHED BY S. HIGHLEY, 32, FLEET STREET,

*Re-printed in New York, by Mr. Wood.*

1840.

W1
ME 75 T

PRINTED BY F. HAYDEN,
Little College Street, Westminster.

### INDEX.

Abdominal tumor, case of.......... 505
Abortion, Mr. Streeter on.......... 178
Abscess, hepatic ....... .......... 535
Absurd practice in Parisian hospitals 193
Academie Royale de Medecine ...... 415
Action of tobacco ................ 344
Acute pleuropneumonia, statistics of 428
Acute hydrocephalus, Dr. Davis on .. 465
Acute ophthalmia ............. ... 476
Adipose tumors .................. 372
Adipose cysts ................... 373
Adipose tumor, laminated.......... 373
Affections of the cornea, inflammatory 54
Affections of the membrana tympani 78
Affections of the mucous membrane, nitrate of silver in ...... ....... 273
Africa, Western, military report from 2
Age, influence of, on mortality among troops .......... ............. 46
Agues, influence of splenic engorgements on..................... 196
Air, introduction of, into the veins .. 275
Albuminous urine, Dr. Bright on.... 251
Albuminous urine, cases of dropsy with 252
Albuminous urine in connection with scarlatina .................... 254
Albuminous urine, Dr. Barlow on.... 263
Algoa Bay ...................... 29
Alison on inflammation ............ 83
Amaurosis, functional ........... 242
Ammon on iritis.................. 512
Ammoniacal pommade, M. Trousseau on 203
Amnion, dropsy of the ........... 99
Amputations, statistical reports of .. 551
Amputations, mortality after ...... 552
Amputations, statistics of.......... 559
Analyses of mineral waters at Brighton 569
Analysis of tobacco .............. 343
Anatomical observations on glands .. 191
Anatomist's vade-mecum, Mr. Wilson's 580
Anatomy of suicide, Mr. Winslow's.. 161
Anatomy, comparative of the nervous system............... ........ 173
Anatomy of glands .............. 190
Anatomy, studentships in .......... 283
Andral on experiments ............ 508
Antimony, fatal effects of tartrate of 215
Apoplexy of the lungs ............ 496
Arborescens lipoma .............. 373
Army, Belgian, ophthalmia in the.... 207
Arsenic, peroxide of iron, in poisoning by 534
Arthritic ophthalmia .............. 59
Artificial nipple of ivory .......... 535
Ashwell on undue lactation ........ 241
Asphyxia, Mr. Lucas on............ 148
Auscultation, Dr. Benson on........ 150
Auscultatory signs of phthisis ...... 494
Avulsion, Dr. Macfarlane on........ 150

B.

Baillarger on the cerebral convolutions 426
Balanitis, Mr. Johnson on.......... 152
Balanitis, causes of................ 153
Balanitis, treatment of ....... .... 153
Bandage, Mr. Chapman on ........ 154
Barbarous operation for fistula ...... 549
Barlow on albuminous urine........ 263
Bauden's on glandular swellings .... 220
Benson on auscultation ............ 150
Berlin, medical relief at............ 358
Berlin, hospitals at................ 359
Bethlem Hospital, report from ...... 545
Blepharitis ...................... 51
Blepharitis, purulent of infants ... 51
Blisters, Mr. Ure on .............. 154
Blood, milky, cause of ............ 532
Bloodletting, Mr. Wardrop on ...... 155
Bodies, preservation of, for dissection 536
Bone, diseases of, Mr. Wells on .... 155
Bones, enchondroma in the ........ 364
Bottex on insanity ................ 218
Brain, Law on disease of the ...... 279
Bread, unfermented .............. 284
Breast, cancer of the, in a male .... 556
Breasts, inflammation of the....... 112
Breschet on glanders .............. 231
Bright on renal disease ............ 251
Bright's kidney the cause of albuminous urine .................... 563
Brodie, Sir B. testimonial to........ 285
Bronchotomy, Mr. Wells on........ 155
Bubo, Mr. Johnson on ............ 156
Bubo in a healthy habit............ 156
Bubo in a scrofulous habit.......... 157
Bubo in a cachectic habit .......... 158
Bubo, treatment of ... .......... 158
Bubo, indurated, local treatment of.. 159
Bubo, suppurating, treatment of .... 159
Burns and scalds, Mr. Ure on ...... 160

C.

Cæsarian operation, cases of ........ 521
Calcutta, cases of hydrocele at...... 284
Campbell on extra-uterine gestation.. 175
Cancer, Dr. Muller on ............ 119
Cancer, Muller on ................ 362
Cancer, Dr. Walshe on ............ 362
Cancer, nosological position of ...... 381
Cancer, physiology of.............. 383
Cancer, effects of, on adjoining parts 385
Cancer, mortality from ............ 392
Cancer of the breast ...... ...... 556

Cape Coast Command, medical report 12
Cape of Good Hope .............. 22
Cape of Good Hope, mortality at the 24
Cape of Good Hope, diseases of the.. 25
Capuron on convulsions during pregnancy .......................... 199
Carbonate of iron as an antidote to arsenic........................... 592
Carcinoma simplex vel fibrosum .... 131
Carcinoma reticulare .............. 133
Carcinoma alveolare .............. 135
Carcinoma melanodes............... 136
Carcinóma medullare............... 137
Carcinoma fasciculatum .......... 140
Carcinoma, development and softening 141
Carcinoma, chemical properties of .. 142
Carcinoma, nature of............... 143
Carcinoma no heterologous structure 144
Carcinomatous growths, minute structure of........................... 129
Carcinomatous growths in general .. 129
Cartilaginous tumors ............... 362
Catarrhal ophthalmia ............ 478
Caudate corpuscules, development of 128
Cazenave on scabies ............... 198
Ceely on the variolæ vaccinæ ...... 408
Cerebellum, softening of both lobes of 562
Cerebral convolutions, Dr. Baillarger on 426
Chapman on bandaging............. 154
Chemosis ......................... 480
Chilblains treated with mustard baths, 200
Child-bed, diseases incident to...... 109
Cholesteatoma ................... 373
Cholesteatoma, general structure of .. 374
Cholesteatoma, microscopic structure 374
Cholesteatoma, chemical relations of.. 375
Cholesteatoma, seats and forms of .. 375
Cholesteatoma in cysts ..... ..... 376
Cholesteatoma on the surface of ulcers 376
Cholesteatoma, development of...... 376
Choleateatoma, nature of .......... 376
Chorea, cimicifuga in the treatment of 282
Churchill on diseases incident to pregnancy ........................... 95
Cimicifuga in the treatment of chorea 282
Circulating system, disorders of the.. 102
Civiale on the grey deposit in urinary calculi ........................... 204
Cleland on tobacco................. 332
Club-foot, M. Guerin on ........... 226
Colchicum, fatal effects of.......... 215
Compound cystoids and cysts ...... 377
Compound cystoid growths ........ 377
Conjunctiva, chronic inflammation of 484
Connections of the placenta........ 237
Constitutional consequences of pregnancy .......................... 96
Convalescence of puerperal women .. 109
Convulsions during pregnancy ...... 199
Cooper on the treatment of stricture 244
Cornea, inflammatory affections of .. 54
Cornea, coloration of the .......... 54
Costello's Cyclop. of Practical Surgery 148
Crania Americana, by Dr. Morton.... 434
Cure of squinting ................ 282
Cutaneous transplantations ........ 200
Cutaneous disease, Dr. Willis' illustrations of ....................... 584
Cyclopædia of Practical Surgery, 148, 362
Cysto-sarcomatous growths ........ 378
Cysts, adipose..................... 373

D.

Davis on hydrocephalus............ 465
Deafness, treatment of .. ......... 221
Development of morbid growths .... 127
Development of caudate corpuscules 128
Development of carcinoma ....... 141
Development of enchondroma ...... 368
Diagnosis, facility of, in general .... 211
Diagnosis, difficulty of, in certain cases 211
Diseases prevalent a Sierra Leone.... 6
Diseases of the ear, Dr. Williams on 73
Diseases incident to pregnancy, Dr. Churchill on ................... 95
Diseases from sympathetic irritation.. 100
Diseases incident to childbed........ 109
Diseases of the mamma, Lisfranc on.. 194
Diseases of old age................ 415
Diseases of the eye, Tyrrell on...... 471
Dislocation of the shoulder joint .... 249
Disorders of the circulating system.. 102
Disorders of the nervous system .... 103
Disorders of the respiratory system .. 103
Dissection, preservation of bodies for 536
Donovan on the hydrocyano-ferrate of quinina ................... ... 566
Donovan on cod oil ............... 566
Dracunculus, Mr. Clarke's case of .. 585
Dreadnought Hospital Ship ........ 549
Dropsy of the amnion.............. 99
Dublin hospitals................... 71
Duodenum, rupture of, &c. ........ 586
Dyspnœa......................... 103

E.

Ear, Dr. Williams on the .......... 73
Eastern Frontiers District, report from 21
Elbow-joint, Key on excision of the.. 247
Emphysema, traumatic ............ 561
Empyema, precautions in operating for 560
Enchondroma............. ...... 362
Enchondroma in the bones ........ 364
Enchondroma, microscopic examination of....................... 364
Enchondroma, chemical analysis of .. 365
Enchondroma of the soft parts...... 368
Enchondroma, nature of .......... 369
Enchondroma, local causes of ...... 369
Enchondroma, general causes of .... 369
England and France, ophthalmic surgery in......................... 49
England, medical tour in .......... 62
English obstetricy, remarks on...... 223

Epidemic hemeralopia............... 223
Epilepsy, oxide of silver in ........ 296
Epileptic convulsions.............. 105
Europe, importation of tobacco into 338
Eustachian tube, inflammation of the 80
Exanthematous ophthalmia ........ 487
Excision of the elbow-joint ........ 247
Excision of the elbow-joint ........ 549
Experiments, M. Andral on ........ 508
Extra-uterine gestation, Mr. Campbell on........................ 175
Eye, Dr. Franz on the ............ 184
Eye, Tyrrell on diseases of the...... 471
Eye, examination of the............ 472
Eye, treatment of diseases of the.... 473

F.

Facility of diagnosis in general...... 211
Fallacies in surgical works ......... 211
Fatal effects of colchicum, &c. ...... 215
Femur, fractures of the neck of the.. 564
Femur, congenital subluxations of the 520
Fever, flaccid state of the ....... .. 562
Fistula, barbarous operation for ..... 549
Fracture, ununited................. 553
Fractures, weight and pulley for .... 47
France and England, ophthalmic surgery in ....................... 49
France, new system of weights in.... 529
Franz on the eye .................. 184
Frequency of the pulse in infants.... 512

G.

General management of pregnant women ......................... 97
Genital organs, diseases of in pregnancy 97
Gestation, existence of milk in urine during ......................... 270
Gipsies metamorphosed........... .. 334
Glanders, Rayer, and Breschet on.... 231
Glands, natural order of............ 190
Glands, intimate structure of........ 191
Glands, anatomy of .............. 191
Glandular swellings, M. Baudens on.. 220
Guerin on subcutaneous wounds .... 225
Guerin on club-foot, wry neck, &c... 226
Guerin on subcutaneous orthopædy.. 519
Guerin on subcutan. incision of joints, 519
Guthrie on the division of the rectus, 537
Guy's hospital reports ............ 241

H.

Hæmatemesis ..................... 101
Hæmitis, M. Piorry on ............ 501
Hæmoptysis ...................... 103
Hamstring tendons, divisions of the.. 281
Health of the navy................. 320
Heart, palpitation of the......... .. 102
Heart, M. Pigeaux on diseases of the, 493
Heart, flaccid state of the, in fever .. 562
Heart, rupture of, into pericardial sac, 571
Hemeralopia, epidemic ............ 223
Herniæ, M. Malgaigne on .......... 229
Herniæ, statistics of .............. 230
Hingeston on dislocation of the shoulder-joint ........................ 249
Hip-joint, injury of the............ 555
History and property of tobacco .... 332
History of tobacco................ 334
Holland and Germany, operative midwifery in ....................... 521
Hospitals in London .............. 66
Hospitals, clinical instructions in.... 69
Hospitals, Dublin ................. 71
Hospitals of Malta ................ 280
Hottentot troops ................. 33
Hudson on the use of nitrate of silver, 273
Hydrocele, cases of, at Calcutta .... 284
Hydrocephalus, Dr. Davis on........ 465
Hydrocephalus, symptoms and diagnosis of ....................... 465
Hydrocephalus, predisposing causes of 466
Hydrocephalus, exciting causes of .. 467
Hydrocephalus, proximate cause .... 467
Hydrocephalus, treatment of........ 468
Hydrocyanoferrate of quinina, Donovan on the ..................... 566
Hydropthalmie, successful treatment of 274
Hysteritis ....................... 99

I.

Iceland, diseases of................ 424
Imperforate vagina, case of ........ 568
Importation of tobacco into Europe.. 338
Incontinence of urine in pregnancy .. 107
Incontinence of urine in old age .... 513
Infantile remittent fever, Dr. Locock on 87
Inflammation of the Eustachian tube, 80
Inflammation, Dr. Alison on........ 83
Inflammation of the breasts ........ 112
Inflammatory affections of the cornea 54
Influence of age on the mortality among troops ......................... 46
Injuries of the hip-joint............ 555
Insanity, abolition of restraint in.... 542
Insanity, M. Bottex on ............ 218
Involuntary seminal discharges...... 533
Iodide of potassium, death from .... 550
Ioduret of iron for syphilitic ulcers .. 534
Iritis, M. Ammon on.............. 512
Iron, peroxide of, in poisoning by arsenic... ..................... 534
Ivory, artificial nipples of .......... 535

J.

James' retrospective address in surgery 47
Johnson on balanitis .............. 152
Johnson on bubo ................. 157
Journal of a medical tour in England, 62

K.

Keratitis ....................... 54
Keratitis, sequelæ and complications of 57
Key on excision of the elbow-joint .. 247
Knee, wound of the .............. 248

L.

Labia, œdema of the .............. 97
Labia, sanguineous tumor of the .... 113

Lactate of iron, remedial power of the 511
Laminated adipose tumor .......... 373
Lane on the oxide of silver ......... 289
Law on disease of the brain ........ 279
Lecanu on the urine ............... 432
Leech gathering in Russia .......... 573
Leucorrhœa, vaginal ............... 98
Library of Practical Medicine........ 81
Ligamentum patellæ, rupture of the.. 554
Lipoma ... ........ ............ 372
Lipoma simplex ................. 372
Lipoma mixtum .................. 373
Lipoma arborescens ............. 373
Lisfranc on medicine and surgery.... 193
Lisfranc on diseases of the mamma .. 194
Lisfranc on fallacies in surgical works, 211
Local consequences of pregnancy.... 96
Local paralysis, German treatment of, 535
Locock on infantile remittent fever .. 87
Locock on puerperal fever.......... 92
London hospitals ................. 66
Loss Island, mortality at .......... 11
Lower jaw, rigidity of the.......... 567
Lucas on asphyxia ................ 148
Lungs, apoplexy of the ............ 496
Lungs, state of the, in hooping-cough 564

M.

Macfarlane on evulsion ............ 150
Magnetisme animal, manual pratique, 575
Malformation of œsophagus ........ 569
Malgaigne on herniæ .............. 229
Malta, hospitals of ............... 280
Mamma, Lisfranc on diseases of the.. 195
Massachusett's general hospital...... 559
Mastodynia ..................... 106
Maternal management of children, Dr. Bull on......................... 579
Mauritius, medical report from the .. 35
Mauritius, diseases of the .......... 39
Meatus auditorius, examination of the 74
Mechanical pressure, disorders arising from......................... 107
Medical tour in England .......... 62
Medical meteorology .............. 212
Medical statistical report, naval ..... 297
Medical relief in Rome and Prussia .. 345
Medical puffing .................. 510
Medical Association, Ireland, resolutions of ...................... 572
Medicine, new terms in ........... 48
Medicine, Practical, Library of ...... 81
Medicine and surgery, Lisfranc on .. 193
Membrana tympani, affections of the.. 78
Membrana tympani, ulceration of the, 78
Memoires of the Academie Royale de Medecine...................... 415
Meredith on parochial medical relief, 168
Microscopic charac. of morbid growths 124
Military medical report. Tulloch's.. 1
Milk, existence of, in the urine during gestation ..................... 270
Milky blood, cause of.............. 532
Mineral waters, analyses of ........ 569
Minute structure of carcinoma ...... 129
Monæsia, notice sur le ............ 174
Montrose lunatic asylum ... ...... 542
Morbid growths, uncertain external character of...................... 119
Morbid growths, chemical examinations of ........................ 123
Morbid growths, gelatinous ........ 123
Morbid growths, albuminous........ 123
Morbid growths, microscopic characters of ........................ 124
Morbid growths, development of .... 127
Mortality at Sierra Leone .......... 6
Mortality at the Cape.............. 24
Mortality of troops in the Mauritius, 36
Mortality among troops, influence of age on ......................... 46
Mortality of officers .............. 46
Morton's Crania Americana ........ 434
Mucous blepharitis................ 52
Müller on cancer............... 119, 362
Müller on secreting glands.......... 189
Müller's elements of physiology ..... 581
Murphy, Dr. his reclamation........ 594
Mustard-baths for chilblains........ 200

N.

Nature of enchondroma............ 369
Naval medical statistical report...... 297
Naval med. officers, advantages to ... 573
Necrosis ....................... 48
Nervous system, disorders of the .... 102
Nervous system, comparative anatomy of the ........................ 173
New terms in medicine ............ 48
New operation for tapping.......... 548
Nitrate of silver in diseases of the mucous membrane............... 273
Norris' report of surgical cases...... 553
Notice sur le monæsia ............ 174
Novel monstrosity .. ............. 516

O.

O'Beirne on the treatment of hydropthalmie ......... ......... .... 274
Obstetric medicine, Dr. Ramsbotham's 286
Œdema of the labia .............. 97
Œsophagus, malformation of........ 569
Officers, mortality of .............. 46
Old age, diseases of................ 415
Operation for stone ............... 419
Operation for prolapsus recti........ 515
Operation for the cure of squinting .. 537
Operative surgery of tumors........ 275
Operative midwifery in Holland and Germany... ............... .. 521
Ophthalmia in the Belgian army .... 207
Ophthalmia, purulent, of children.... 209
Ophthalmia, simple acute .......... 476
Ophthalmia, pustular.............. 478
Ophthalmia, purulent.............. 479

Ophthalmia, exanthematous ........ 487
Ophthalmic surgery in France and England ..................... 49
Orfila on poisoning.... ........... 428
Orthopædy, subcutaneous, M. Guerin 519
Otitis ......................... 75
Otorrhœa ...................... 76
Oxide of silver, Lane on the ........ 289

P.

Palm of the hand, wound of the .... 554
Paracentesis thoracis, suggestion in.. 492
Parochial med. relief, Mr. Meredith on 168
Pathological facts . .............. 561
Penny postage, benefits of the ...... 545
Pharmacopée universelle .......... 580
Phlebitis, uterine . .............. 114
Phthisis, auscultatory signs of ...... 494
Physiological corollaries on secretion 192
Physiology of ventriloquism ........ 527
Pigeaux on diseases of the heart .... 493
Piorry on hæmitis ................ 501
Placenta, structure and connections of 237
Pneumonia, statistics of............ 491
Pommade ammoniacale, M. Trousseau on the ....................... 203
Poor in Prussia, medical relief of the 345
Practical medicine, library of ...... 81
Practical surgery, Cyclopædia of .... 148
Precaution in operating for empyema 561
Pregnancy, consequences of ........ 96
Pregnancy, Churchill on diseases incident to ..................... 95
Pregnancy, menstruation during .... 98
Pregnancy, convulsions during...... 199
Pregnant females, treatment of...... 97
Pregnant females, diseases of the genitals in ....................... 97
Priessnitz' sweating regimen ........ 523
Professional aphorisms ............ 531
Prolapsus recti, new operation for .. 515
Provincial Med. and Surg. Association, Transactions of the............... 397
Pruritus of the vulva .............. 98
Prussia, Medical Relief in.......... 345
Puerperal fever, Dr. Locock on...... 92
Puerperal women, convalescence of .. 109
Puerperal fever ................. 114
Puerperal peritonitis .............. 114
Puerperal hysteritis .............. 114
Puerperal mania ................. 118
Pulmonary consumption, Mr. Bodington on ............... ........ 581
Pulse, frequency of the, in infants .. 512
Purulent ophthalmia ....... ...... 53
Purulent ophthalmia of children .... 209
Purulent ophthalmia.............. 479
Purulent ophthalmia in the adult .... 479
Purulent ophthalmia in the infant .. 483
Pustular ophthalmia .............. 478
Pyrosis......................... 101

Q.

Quain's plates ..... .............. 580
Queen's hospital, Birmingham, address 578

R.

Radial artery, ligature of the........ 554
Ramsbotham on obstetric medicine .. 285
Rare cases, on the study of ........ 547
Rayer on glanders ... ............ 231
Rectus muscle, Guthrie on the division of the ....................... 537
Rees on the proportions of urea .... 268
Remarks on stammering .......... 489
Remedial power of the lactate of iron 511
Reminiscences of Rome .......... 345
Renal disease, Dr. Bright on ........ 251
Report of the vaccination section .... 397
Resolutions of Medical Association of Ireland ... ................. 572
Respirator ..................... 286
Respiratory system, disorders of the.. 102
Restraint in insanity, abolition of.... 542
Retrospective address in surgery ... 47
Re-vaccination ................. 404
Revulsion ..................... 81
Rigidity of the lower jaw .......... 567
Rome and Prussia, medical relief in.. 346
Rome, reminiscences of............ 345
Rome, hospitals in ................ 346
Rome, medical administration of the hospitals in ..................... 347
Rupture of the ligamentum patellæ .. 554
Rupture of the heart into pericardial sac ......................... 571
Roux' operation for fistula....... .. 549

S.

St. Helena, state of troops at........ 1
St. Helena, medical report from .... 17
St. Helena, diseases of ............ 19
Salivation ..................... 100
Sanatory effects of tobacco ........ 344
Sanguineous tumor of the labia .... 113
Sarcomatous growths.............. 377
Scabies, M. Cazenave on .......... 198
Scarlatina, connection of albuminous urine with ........... ........ 254
Sclerotitis ..................... 58
Scrofulous ophthalmia ............ 485
Scrofulous ophthalmia, chronic...... 486
Scrophulous ophthalmia.... ....... 50
Seamen, employment of............ 301
Seamen, berthing of .............. 302
Secreting glands, Muller on ........ 189
Secretion, physiological corollaries on 192
Shoulder-joint, dislocation of ...... 249
Sickness, &c. of Troops in Western Africa ...................... 1
Sickness, &c. of troops in St. Helena.. 1
Sickness, &c. of troops at the Cape of Good Hope .................... 1
Sickness, &c. of troops at the Mauritius 1

Sierra Leone .................... 2
Sierra Leone, climate of............ 3
Sierra Leone, mortality of troops at.. 6
Sierra Leone, diseases of............ 7
Skin, Dr. Thomson on diseases of.... 583
Small-pox and cow-pox, affinities between ........................ 398
Small-pox after small-pox.......... 403
Smart on division of the ham-string tendons ...................... 281
Solutio magnesiæ bicarbonatis ...... 597
South American station............ 307
Sphincter, removal of, for prolapsus ani 573
Splenic engorgements, influence of, on agues ......................... 196
Squinting, cure of ................ 281
Squinting, operation for the cure of.. 537
Stammering, remarks on .......... 489
Statistics of pneumonia ............ 491
Statistics of amputations .......... 559
Stevens on tumors ................ 275
Stomach and duodenum, cramps of the 101
Stone, operation for .............. 419
Streeter on abortion .......... .... 178
Stricture, Cooper on the treatment of 244
Studentships in anatomy .......... 283
Study of rare cases................ 547
Subcutaneous wounds, M. Guerin on 225
Subcutaneous orthopædy, M. Guerin on 519
Subcutaneous incision of joints, M. Guerin on .................... 519
Subluxations of the femur.......... 520
Suggestion in paracentesis thoracis .. 492
Suicide, Winslow's anatomy of ...... 161
Surgical works, fallacies in ........ 211
Surgical cases. Norris ............ 553
Surgery, retrospective address in .... 47
Surgery, practical, cyclopædia of .... 362
Surgery and Medicine, Lisfranc on .. 193
Swan's comparative anatomy of the nervous system ................ 172
Sweating regimen of Dr. Priessnitz .. 523
Symonds' Dr. retrospective address .. 576
Sympathetic irritation, disorders from 100
Syphilitic ulcers, ioduret of iron in .. 534

T.

Tagebuch einer medicinischen reise nach England .................. 62
Tapping, new instrument for........ 548
Taste, fastidious .................. 100
Testimonial to Sir B. Brodie ........ 285
Tobacco, Dr. Cleland on .......... 332
Tobacco, history of . ............ 334
Tobacco, importation of, into Europe 338
Tobacco, analysis of .............. 343
Tobacco, action of ....... ....... 344
Tobacco, sanatory effects of ........ 344
Toothache ...................... 100
Towns, medical relief in............ 345
Trousseau on pommade ammoniacale 203
Tulloch's military medical report .... 1
Tumor, laminated adipose .......... 373
Tumors, operative surgery of........ 275
Tumors of the scalp, extirpation of .. 275
Tumors, solid, extirpation of........ 276
Tumors, adipose ................ 372
Tweedie's library of practical medicine 81
Tyrrell on diseases of the eye........ 472

U.

Undue lactation, Dr. Ashwell on .... 241
Unfermented bread................ 284
Ununited fracture ................ 553
Ure on blisters .................. 154
Ure on burns and scalds............ 160
Urea, proportion of, in diseased fluids 268
Ureters, spasm of the .............. 107
Urinary calculi, grey deposit on the.. 204
Urine, incontinence of, in pregnancy 107
Urine, retention of, in pregnancy.... 107
Urine, milk in, during gestation .... 270
Urine, Lecanu on the.............. 432
Urine, incontinence and retention of, 513
Uterine appendages, inflammation of 114
Uterine phlebitis.................. 114
Uterine lymphatics, inflammation of 115
Uterus, rheumatism of the ........ 99
Uterus, rupture of the.............. 115

V.

Vaccination section, report of the.... 397
Vaccination, protecting powers of .. 403
Vagina, discharge of watery fluid from 98
Vagina, inflammation of the........ 114
Vagina and uterus, rupture of the.... 115
Vaginal leucorrhœa .............. 98
Vaginal cystocele, new operation for.. 433
Varicose veins.................... 108
Variolæ Vaccinæ, Mr. Ceely on the . 408
Varrentrapp's tour in England .... . 62
Veins, introduction of air into the.... 279
Velpeau on ophthalmic surgery...... 49
Ventilation ...................... 306
Ventriloquism, physiology of........ 527
Vesicant, heat as a................ 204
Vulva, pruritus of the ............ 98

W.

Walshe on cancer ................ 362
Wardrop on bloodletting .......... 155
Weight and pulley for fractures...... 47
Weights in France, new system of.... 529
Wells on the diseases of bones ...... 155
Wells on bronchotomy ............ 155
West Indies and North America .... 320
Western Africa, sickness of troops there 1
Williams on the ear .............. 73
Wilson's naval med. statist. report .. 297
Winslow's anatomy of suicide ...... 161
Wound of the knee................ 248
Wound of the palm of the hand...... 554

### The Respirator.

### BIBLIOGRAPHICAL RECORD.

An Atlas of Plates illustrative of the Principles and Practice of Obstetric Medicine and Surgery. With descriptive Letter Press. By *Francis H. Ramsbotham,* M.D. Member of the Royal College of Physicians, Physician to the Royal Maternity Charity, and Lecturer on Obstetric and Forensic Medicine at the London Hospital, &c. London: J. Churchill, 1840. Parts IV., V., and VI.

This is really a very meritorious, and likely to prove a very useful work. The Parts are published with regularity, and their execution is equal to that of their predecessors. The Plates are extremely good.

The commencement of the Fourth Part is occupied with the description of the decidua, and the ovum with its accompaniments. The description of "Labour" is then taken up and pursued through the succeeding Parts.

We observe that Dr. Ramsbotham doubts, as most anatomists of the present day do, the accuracy of Hunter's view of the mode of formation of the "Decidua Reflexa." For, as he observes, a prolongation of the outer membrane has been frequently observed passing a little way into each Fallopian tube, which could not be the case were the internal merely a duplicature of the outer layer.

We beg to reiterate our favourable opinion of the Work, and to wish its zealous and talented author every possible success.

1. Dictionary of Geology and Mineralogy, comprising such terms in Botany, Chemistry, Comparative Anatomy, Conchology, Entomology, Paleeontology, Zoology, and other Branches of Natural History, as are connected with the Study of Geology. By William Humble, M.D. Octavo, pp. 279. In closely-printed double columns. Washbourne, Salisbury Square, Fleet Street. March, 1840.

2. A Series of Anatomical Sketches and Diagrams, with Descriptions and References. By Thomas Wormald, Assistant Surgeon and Demonstrator of Anatomy at St. Bartholomew's Hospital, and Andrew Melville M'Whinnie, Teacher of Practical Anatomy at St. Bartholomew's Hospital. Quarto, pp. 16. Price 4s. No. 2. Highley, 1840.

3. The India Journal of Medical and Physical Science for August, September, and December, 1840. *In exchange, the India Review for same months*

---

4. Statistical Reports on the Sickness, Mortality, and Invaliding among the Troops in Western Africa, St. Helena, the Cape of Good Hope, and the Mauritius. Prepared from the Records of the Army Medical Department and War-Office Returns. Presented to both Houses of Parliament. 4to, 1840. Presented to the Senior Editor, by Sir JAMES M'GREGOR.

---

5. First Principles of Surgery; being an Outline of Inflammation and its Effects. By GEORGE T. MORGAN, A.M. formerly Lecturer on Surgery in Aberdeen. Part 3, 1840. Highley, Fleet Street. Maclachlan and Stewart, Edinburgh.

---

6. The Phrenological Journal for April, 1840. *In exchange.*

---

7. Odontography; or a Treatise on the Comparative Anatomy of the Teeth, their Physiological Relations, Mode of Development, and Microscopic Structure, in the Vertebrate Animals, illustrated by upwards of 150 plates. By RICHARD OWEN, F.R.S. Hunterian Professor to the Royal College of Surgeons, &c. Part the First, containing seven sheets of letter-press, and fifty plates. London, Bailliere; Paris, Bailliere; Leipzig, Wergel, 1840. Price, plain plates, 1l. 11s. 6d. Quarto proofs, 2l. 12s. 6d.

---

8. Guy's Hospital Reports, No. X. April 1840. Edited by Drs. BARLOW and BABINGTON. Octavo. Highley, 1840.

---

9. A System of Practical Medicine, comprised in a Series of Original Dissertations. Arranged and edited by ALEX. TWEEDIE, M.D. F.R.S. &c. Vol. I. pp. 440. Very close type. Whittaker, 1840.

☞ *This volume contains, "Introduction," Dr. Symonds—"Inflammation," by Dr. Alison—"General Doctrine of Fever," by Dr. Christison—"Continued Fever," Ditto—"Plague," Dr. Shapter—"Intermittent Fever," Ditto—"Remittent Fever," Ditto—"Yellow Fever," Ditto—"Infantile Gastric Fever," Dr. Locock—"Hectic Fever," Dr. Christison—"Small-pox," Dr. Gregory—"Measles," Dr. George Burrows "Scarlatina," Ditto—"Puerperal Fevers," Dr. Locock—"Diseases of the Skin," Dr. H. E. Schedel.*

10. Parochial Medical Relief considered in a letter to the "Poor-Law Commissioners," developing an entirely new System of Medical Remuneration, alike conducive to the Interests of the Rate-Payers, the well-being of the Poor, and the respectability of the Profession. By E. T. MEREDITH, M.R.C.S. Octavo, pp. 35. Price 1s. 6d. Highley, Fleet Street. May, 1840.

---

11. Observations on the Diseases incident to Pregnancy and Childbed. By FLEETWOOD CHURCHILL, M.D, &c. &c. Octavo, pp. 463. Keene and Son, Dublin. May, 1840.

---

12. Memoir on Extra-uterine Gestation. By Dr. WM. CAMPBELL, of Queen's College, Edinburgh, &c. Octavo, pp. 154. Black and Co. Edinburgh; Longman, London. May, 1840.

---

13. An Address delivered at the Anniversary Celebration of the Birth of Spurzheim, and the Organization of the Boston Phrenological Society, December 31, 1839. By GEORGE COOMBE, Esq. Boston, 1840.

☞ *This as might be expected, is an eloquent address, with a dash of enthusiasm.*

---

14. A Letter to Sir Benjamin Brodie, Bart., containing a Critical Enquiry into his "Lectures illustrating certain local Nervous Affections." By WILLIAM GOODLAD, M.R.C.S. &c. &c. Octavo, pp. 154. Longman and Co. May, 1840.

---

15. An Essay on the Treatment and Cure of Pulmonary Consumption. By GEORGE BODINGTON, Surgeon. Duodecimo, pp. 60. Longman and Co. May, 1840.

---

16. A Practical Work on the Diseases of the Eye, and their Treatment, Medically, Topically, and by Operation. By FRED. TYRRELL, Senior Surgeon to the Royal London Ophthalmic Hospital, Surgeon to St. Thomas's Hospital, &c. Two Volumes, 8vo, with plates. Churchill, London. May, 1840.

---

17. A Treatise on the Physiological and Moral Management of Infancy. By AND. COMBE, M.D. Octavo, pp. 374. Maclachlan and Stewart, Edinburgh. 1840.

18. Official Report of the Medical Topography and Climate of Calcutta, with brief Notices of its prevalent Diseases, Endemic and Epidemic. By JAS. RANDAL MARTIN, Presidency Surgeon, and Surgeon to the Native Hospital. Printed by order of Government. Calcutta, 1839.

☞ *We noticed the first edition of this valuable report in our Journal. The present is greatly enlarged and entirely re-constructed and improved. The talented author has returned to his native land, after a hard service of a quarter of a century in India.*

---

19. The Physiognomy of Mental Diseases. By Sir A. MORRISON, M.D. No. 19. Price 3s. 6d. London, May, 1840.

---

20. The Madras Quarterly Medical Journal. Edited by SAMUEL ROGERS, Assistant Surgeon, Madras Establishment. Vol. I. for January, April, July, and October, 1839.

---

21. A Popular View of the Anatomy of the Human Body. By JAMES DOUGLAS, A.M. Surgeon. Duodecimo, pp. 172. Glasgow. May, 1840.

---

22. Vital Dynamics. The Hunterian Oration before the Royal College of Surgeons in London, 14th Feb. 1840. By Jos. HENRY GREEN, F.R.S. &c. Octavo, pp. 125. Pickering, London, 1840.

---

23. The Philosophy of Instinct and Reason. By J. STEVENSON BUSHNAN, M.D. Duodecimo, pp. 316. With Plates. Edinburgh, 1838.

---

24. An Atlas of Plates illustrative of the Principles and Practice of Obstetric Medicine and Surgery; with Descriptive Letter-press. By FRANCIS RAMSBOTHAM, M.D. &c. Nos. 4, 5, and 6. Churchill, London, 1840.

---

25. The Library of Medicine, arranged and edited by ALEX. TWEEDIE, M.D. Vols. II. and III.

---

26. Considerations sur la Structure de l'Encephale et sur les Relations du Crane avec cet Organe. Par le DOCTEUR FOVILLE. Extract de l'Experience, du 16 Avril, 1840.

27. A Practical Essay on Delirium Tremens, written principally with a view to elucidate the division into distinct stages, &c. By ANDREW BLAKE, M.D. Physician to the Nottingham General Lunatic Asylum, &c. Second Edition. Octavo, pp. 112. Longman and Co. 1840.

---

28. Fisher's Historic Illustrations of the Bible. Principally after the Old Masters. Part I. Price 2s. Fisher, Son and Co., Newgate Street. 1840.

☞ *The plates are beautifully executed.*

---

29. Illustrations of Cutaneous Diseases. A Series of Delineations of the Affections of the Skin, in their more interesting and frequent Forms; with a Practical Summary of their Symptoms, Diagnosis, and Treatment, including appropriate Formulæ. By ROBERT WILLIS, M.D. &c. Part XVI. June 1. Price 5s. Bailliere.

---

30. The Transactions of the Provincial Medical and Surgical Association. Instituted in 1832. Vol. VIII. Churchill, London. June, 1840.

---

31. Practical Remarks on the Causes, Nature, and Treatment of Deformities of the Spine, Chest, and Limbs, Muscular Weakness, Weak Joints, Muscular Contractions, and Stiff Joints; containing the Results of the Author's Experience, and shewing the Advantages derived from the Modes of Treatment which he has recently introduced. With illustrative Plates and Cases. By JOSEPH AMESBURY, Surgeon, &c. late Lecturer on Orthopædic Surgery. Quarto, pp. 192. With numerous Plates. Longman and Co. June, 1840.

---

32. Cursory Notes on the Morbid Eye. By ROBERT HULL, Physician to the Norfolk and Norwich Hospital, &c. Octavo, pp. 249. Longman and Co. 1840.

---

33. The Retrospective Address on Surgery, from July, 1836, to July, 1839. Delivered before the Provincial Medical Association at Liverpool, on the 24th July. By S. H. JAMES, Esq. Surgeon to the Devon and Exeter Hospital. Octavo, pp. 92. Worcester, 1840.

